# Interprofessional education in medicine

**DOI:** 10.3205/zma001678

**Published:** 2024-04-15

**Authors:** Marjo Wijnen-Meijer

**Affiliations:** 1 TUD Dresden University of Technology, Medical Faculty and University Hospital Carl Gustav Carus, Institute of Medical Education, Dresden, Germany

## Editorial

Our society’s current demographic development and diversification and the simultaneous persistent undersupply of labour pose new challenges for our healthcare system daily. As a result, healthcare systems are forced more than ever to supplement and replace their multi-professional healthcare provision with interprofessional collaboration [1]. Therefore, in order to be able to master future challenges, current and future students in healthcare professions must be confronted with the topic from early on to avoid uniprofessional training and potential apathy towards team culture [2]. 

Here is precisely where the idea of interprofessional education (IPE) comes in. According to the World Health Organization, IPE “occurs when two or more professions learn about, from and with each other to enable effective collaboration and improve health outcomes” [3].

To concretise the concept of interprofessional education, international efforts have been made to crystallise corresponding frameworks [4], whereby core areas of competence of interprofessional collaboration can be summarised in four major areas with a total of 33 sub-competencies according to the Interprofessional Education Collaborative (IPEC) [5]. IPEC core competence areas revolve around “values and ethics”, “roles and responsibilities”, “communication” and “teams and teamwork”. In practice, healthcare workers encounter these issues in daily practise. It would therefore be desirable for work with team colleagues to be based on shared principles and mutual respect so that complex ethical decisions can be made together. The more precisely roles are defined, boundaries are set and responsibilities are allocated, the better a team will function. Communication in various forms acts as a catalyst for collaboration and can be uniformly improved using established tools [6]. Finally, the team concept includes not only professionals but also patients, families and the local community [7].

In order to prepare for future scenarios, various ideas from the literature are used to strengthen the core competencies of collaboration through teaching. High-fidelity simulations with multiple simulation scenarios [8], active learning sessions through workshops, small group work with case discussions, and role plays are effectively used. Most IPE teaching was done in alternating settings using different modalities and with an alternating mix of students from different professions [9]. IPE activities in a clinical setting and thus at the heart of everyday practice might be more effective than IPE teaching in regular classroom settings [10]. To address the subscale of Roles and Responsibilities, actively interviewing or shadowing health professionals on placement is a common IPE activity and seems highly beneficial [10], [11]. Next, observing team-based meetings for interactions and encouraging student reflection after an IPE activity seems to have increased the outcome measures [11]. 

It is important to mention that even a one-time intervention on IPE can significantly improve the perception of and self-efficacy for IPE, as shown by Jung et al. using, among other things, a role-play simulating a medication-based error [12]. Also, a positive correlation between the number of previous IPE experiences and presurvey IPEC sub competency ratings was found, indicating that previous points of contact with interprofessional collaboration lay the foundation for future IPE learning [13]. Overall, IPE interventions seem to impact attitudes towards professionals of other disciplines, significantly change collaborative behaviour and likely also improve collaborative skills such as communication [14]. Unfortunately, most interventions are shorter than three months and show strong heterogeneity in IPE design and in the assessment of the intervention outcome [8], [14], [15]. Hardly any studies followed a longitudinal approach to assess the impact of IPE incorporation, which makes it difficult to properly develop higher-level complexities of teamwork [9]. 

Based on these existing limitations in the literature, future efforts need to be made to address the following key points. Interprofessional Education should be embedded into academic curricula as well as clinical practice to ensure that interprofessional learning follows a time and knowledge gradient [8]. As academic curricula are set longitudinally, students should be repeatedly exposed to IPEC sub-competencies [13]. It is then just as important to measure corresponding outcomes longitudinally and repeatedly rather than simply comparing pre- and post-survey evaluation scores. Efforts need to be made to reach a general consensus on which tools should be used to evaluate IPE implementation and outcome objectively [15]. Careful preparation of the IPE facilitators will drastically decide the quality of the intervention [7]. Beyond that, psychology seems to be massively underrepresented in the involvement in IPE activities, as the literature about psychology learners being part of interprofessional collaboration interventions is scarce [15]. 

Considering the existing literature, the importance of interprofessional education must be emphasised. Standardised, mandatory and longitudinal development of a rigorous IPE framework, taking into account local specificities, is the most crucial measure to ensure adequate patient care in the face of future challenges. If the problems of the current IPE landscapes listed above do not serve as a wake-up call, interprofessional collaboration's immense potential will probably remain untapped.

Interprofessionalism plays a role in some of the articles in this issue. In her article, Juliette Beuken describes an educational intervention concerning cross-border healthcare. This involves different professions, often with a different division of tasks and responsibilities in different countries [16]. Julia Schendzielorz describes the planning, implementation and evaluation of a longitudinal science curriculum. One of their experiences is that such a curriculum needs the contribution of teachers from different backgrounds, such as epidemiology, anthropology, statistics and public health [17]. With regard to teachers, Franziska Baessler’s article is also relevant. They conducted a study on what kind of didactic training physicians and psychologists need [18]. In addition to several research articles, this issue contains the position paper of the GMA Committee “teaching evaluation”. This contains recommendations for the further development of evaluation [19]. 

Hopefully this issue will provide new inspiration!

## Competing interests

The author declares that she has no competing interests.
